# Bayesian geochemical correlation and tomography

**DOI:** 10.1038/s41598-024-59701-4

**Published:** 2024-04-23

**Authors:** Hugo Bloem, Andrew Curtis

**Affiliations:** School of Geosciences, University of Edinburgh, Edinburgh, EH9 3FE UK

**Keywords:** Geochemistry, Geology, Geophysics, Palaeontology, Geochemistry, Palaeoclimate

## Abstract

To accurately reconstruct palaeoenvironmental change through time it is important to determine which rock samples were deposited contemporaneously at different sites or transects, as erroneous correlation may lead to incorrectly inferred processes and rates. To correlate samples, current practice interpolates geological age between datable units along each transect, then temporal signatures observed in geochemical logs are matched between transects. Unfortunately spatiotemporally variable and unknown rates of sedimentary deposition create highly nonlinear space-time transforms, significantly altering apparent geochemical signatures. The resulting correlational hypotheses are also untestable against independent transects, because correlations have no spatially-predictive power. Here we use geological process information stored within neural networks to correlate spatially offset logs nonlinearly and geologically. The same method creates tomographic images of geological age and geochemical signature across intervening rock volumes. Posterior tomographic images closely resemble the true depositional age throughout the inter-transect volume, even for scenarios with long hiatuses in preserved geochemical signals. Bayesian probability distributions describe data-consistent variations in the results, showing that centred summary statistics such as mean and variance do not adequately describe correlational uncertainties. Tomographic images demonstrate spatially predictive power away from geochemical transects, creating novel hypotheses attributable to each geochemical correlation which are testable against independent data.

## Introduction

Geochemical signatures recorded in stratigraphic columns of sedimentary rocks form a primary data source concerning palaeoenvironmental conditions through time^1^. However, the temporal record along any stratigraphic transect usually contains gaps due to depositional hiatuses, so datasets from different spatial locations must be combined to form more complete time series^2^. Observations of similar signatures on contemporaneous, spatially disparate stratigraphic transects also allows local and regional environmental conditions to be discriminated. Matching samples deposited contemporaneously on different geochemical data logs, a step referred to as *correlation*, is therefore key to making robust palaeoenvironmental interpretations.

Absolute ages of sediments are only available for samples in lithologies conducive to radiometric or other dating methods which are often absent over large sections of a stratigraphic column. Correlation therefore requires temporal interpolation of ages to all other samples on each transect^3^. Most studies assume piecewise linear relations to convert from space to time^4^, even though this relationship is known to be highly nonlinear due to hiatuses and variations in deposition rate^5,6^.

Existing correlation methods and algorithms focus mainly on pattern matching—varying the space-time transformation to improve the visual or numerical match between coeval patterns observed in data logs from different transects^2,7,8^. Experts who apply these methods are able to find correlations between some sets of logs, but pattern-matching methods tend to fail if data from the same time interval differ significantly between logs^6^. Correlations are therefore always in error to some extent. Unfortunately, they do not readily submit to hypothesis testing against data observed on independent transects, because correlations between existing transects have no predictive power elsewhere. That is, current methods provide little information about the space-time conversion in the inter-transect volume, as correlation is performed either directly in log height or in a pseudo-time domain constructed along each transect.

Uncertainties quoted in geochemical stratigraphy are often limited to geochemical measurement uncertainties while correlation uncertainties remain unquantified^9,10^. Bowyer et al.^11^ address correlation uncertainties by presenting multiple possible correlations.^12^ and^13^ each present different correlations for the same logs. However, due to the nonlinearity in the true space-time conversion, many correlations may be valid to some level of certainty^8^, yet none of these studies quantify the relative probability of different possible correlations. A Bayesian method that estimates uncertainty was implemented by Eichenseer et al.^14^, but it contains an implicit assumption that synchronous geochemical signatures in all transects should match, and the method has no spatially predictive power.

This work introduces a probabilistic method for converting from space to time by adding dynamic geological process information to the correlation process. This allows geochemical signals that manifest as different patterns between logs to nevertheless be correlated correctly, provided that the logs can be predicted by a known geological or geochemical process. The method also images the geochemical signature or stratigraphic age of sediments tomographically in the volume of rock around the transects, in particular in the inter-transect rock volume, and estimates full Bayesian uncertainty on all results. Results can therefore be treated as hypotheses to be tested against data from independent transects in the imaged rock volume by comparison with the tomographic image.

Sedimentary geological process modelling (GPM) involves computational simulation of geological processes over geological timescales to produce three-dimensional virtual geologies. We use many such simulations to infuse our method with information about dynamic geological processes. GPM initiates from a particular time and base topography, and simulates variations in sea level and the 3D distribution of sedimentary deposition, erosion, transport and redeposition, in addition to a variety of other model-dependent processes^15–22^. The age of deposition and the sediments preserved at each 3D location are predicted, and by considering variations in the chemistry of the marine water it is possible to simulate the geochemical signature throughout the preserved stratigraphy.

A disadvantage of GPM is computational cost, and the difficulty involved in fitting the models to specific observations from logs or geological outcrops^23,24^. We overcome these difficulties by training a generative adversarial network (GAN) to predict space-time transforms from many GPM results. This allows Bayesian inference to be applied to correlate intra-basin logs while implicitly accounting for information about dynamic geological processes. Fully nonlinear Bayesian methods have been applied in geophysical tomographic applications in recent years^25–27^, but only recently have they incorporated dynamic geological information^28–30^. However, no published work uses geochemical data and GPM for Bayesian correlation, and neither have geochemical data been used for inter-transect tomography. In principle, Bayesian inference provides a probability distribution over all possible correlation and tomographic models that are consistent with observations and pre-existing geological knowledge. The non-uniqueness of geochemical correlations demonstrated in previous work makes this especially important.

The $\delta ^{13}$C isotope ratio relates the sedimentation of organic carbon to the total carbon^31^ and is often used as a proxy for biological activity over geological timescales^1,32^. We demonstrate the method using synthetic $\delta ^{13}$C datasets.

## Methods

We first discuss dynamic process modelling of geological data and the creation of space-time transforms, then a method to embody this information within neural networks, and finally we combine these to create a Bayesian method to perform correlation of logs and geochemical tomography.

### Geological information

The conversion of geochemical observations from space to time would be simple under conditions of constant sedimentation rate and no erosion, as is often assumed between interpreted hiatuses in conventional correlation methods^2^. In reality the interaction of dynamic processes typically results in a relationship that is strongly nonlinear and spatially variable. Logs are usually recorded along sub-vertical transects and this variability causes the height-to-time relationship to vary with location—for example, logs from samples deposited in deeper water tend to conserve more of the geochemical record compared to those from shallower areas^6,33^. We constrain the space-time relationship using geological information derived from GPM software *SedSimple*, which simulates geological processes of sedimentary deposition, erosion, transport and redeposition to produce a synthetic stratigraphy in space and time^20^. Computational simulations provide complete information about the time of deposition of sediment preserved at any location, and the temporal length of every hiatus, so each simulation produces a spatio-temporally complete space-time transform.

**Figure 1:**
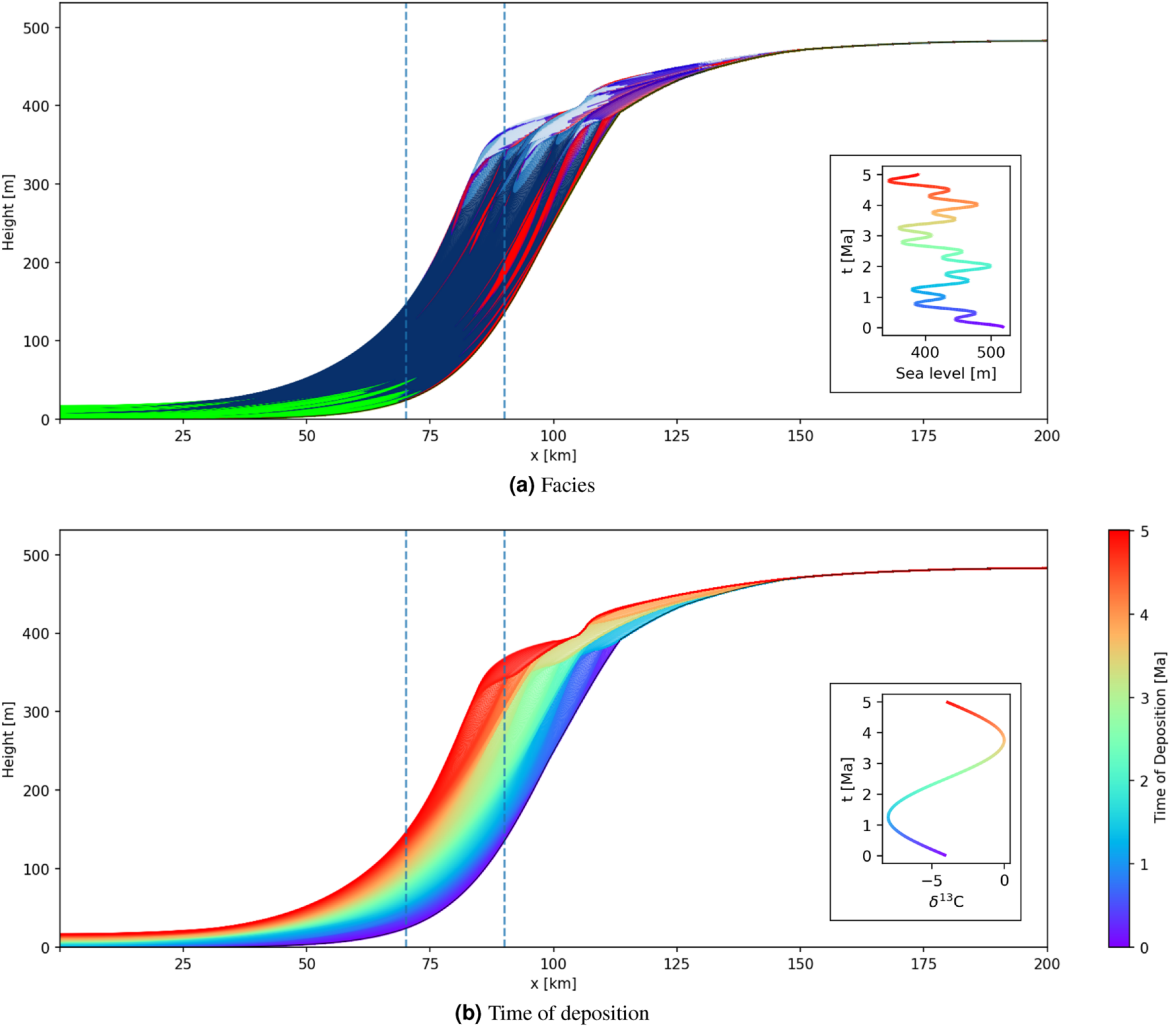
Cross-sections through an example GPM simulation from software package *SedSimple* showing (**a**) a facies map where red and green colours represent coarse and fine siliciclastic respectively, blue represents carbonates. Colours grade between these canonical facies to reflect the relative volumes of each in mixed facies. (**b**) The time of deposition (equivalent to geological age) of facies preserved at each location. The vertical lines show transect locations in Fig. 4a. Inset panel (**a**) shows the sea level curve imposed during the simulation, and inset (**b**) shows the secular change of $\delta ^{13}$C in marine water chemistry used to derive geochemical signatures in Fig. 4a. The colourmap of both insets represents the time of deposition along the transect in panel (**b**).

**Figure 2:**
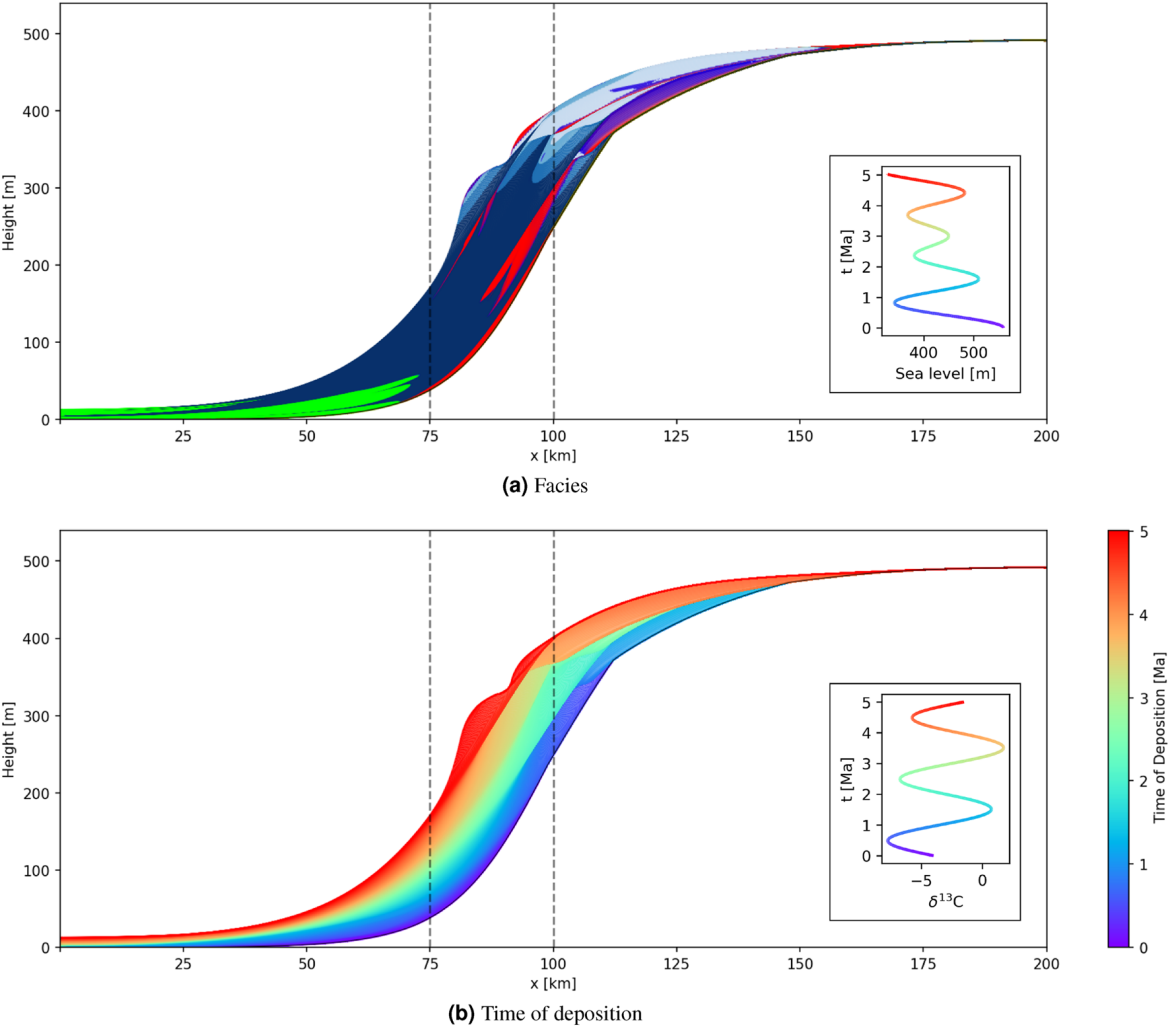
Similar to Fig. 1 but for a GPM simulation with different sea level oscillations and secular variations in sea water geochemistry. Vertical lines correspond to the transect locations in Fig. 4b.

Figures 1 and 2 show 2D basin-to-land cross-sections through 3D volumes produced by two example GPM simulations, referred to as geological model A and B, respectively. Panels (a) show geological facies represented by different colours: red and green represent coarse and fine siliciclastics respectively, blue represents carbonates. Panels (b) display the time of deposition at each location in the preserved sediment and is therefore exactly the space-time transform produced by this simulation. The two simulations differ only in the pattern of sea level variations as shown in the inset panels (a).

Vertical geochemical transects are simulated at horizontal locations 70 km and 90 km through model A, and 75 km and 100 km through model B, by assuming that sediments record the secular variation in seawater chemistry at time of deposition, shown in the figure insets. Transects through geological model A are chosen to pass through deeper water and therefore preserve more of the geochemical signature than those through model B, so in principle it may be easier to correlate the former than the latter.

### Storing geological information in neural networks

Simulating geological processes with GPM is computationally expensive—a single simulation can take days to run. This is problematic because for Bayesian correlation many models must be generated and tested against observed geochemical signatures. We therefore train a generative adversarial network (GAN) to generate models resembling those from GPM; generating such models is then possible in under a second, albeit with slightly less detail and accuracy^30,34–38^ (see below for details about network structure and training). The GAN is trained to represent a mapping from a low-dimensional latent space of random variables to the high-dimensional geological model space. The latent variables have arbitrary, user-defined continuous probability distributions but have no intuitive meaning. The GAN nevertheless translates any set of values of the latent parameters into a geological model sample; since each such sample includes a space-time transform it can be used to convert logs from height to age of deposition.

The abstract nature of the sampling process makes it almost impossible for a human to find geological models that fit observed data to within their uncertainties on all transects. Furthermore, while the latent space is relatively low-dimensional it still has 30 parameters (dimensions) to explore, creating a parameter space which is densely packed with information. As a result, continuous changes in any combination of latent parameters causes the model to update continuously, but highly nonlinearly. Algorithms used for Bayesian inference are therefore designed to explore the latent space by analysing many models, to find those that produce satisfactory data fits^39^.

**Figure 3:**
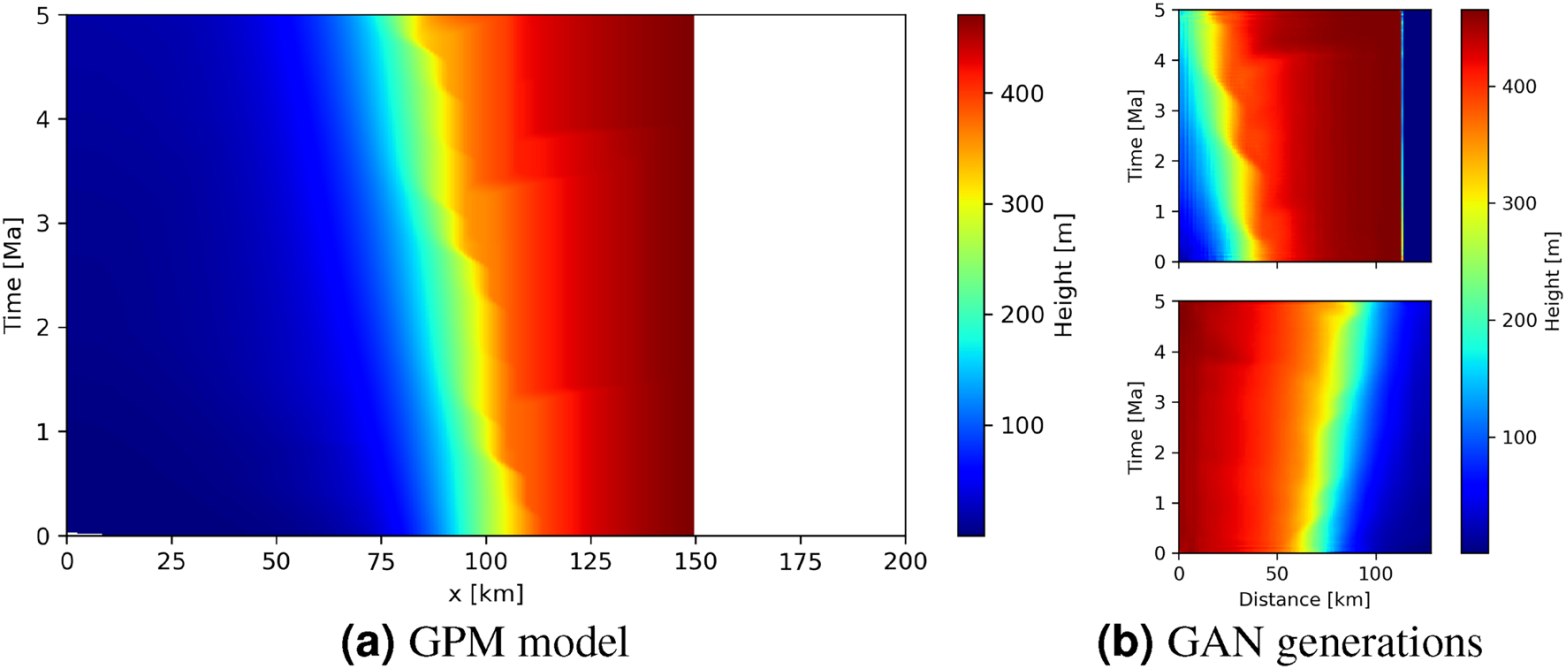
(**a**) An alternative view of the space-time transform in Fig. 1b. Here, the y-axis represents the time of deposition and the colourmap shows the corresponding height on any vertical transect through geological model A at horizontal offset *x*. The white region indicates that no sediment was deposited for $x>$ 150 km. (**b**) shows two examples of GAN generated samples of space-time transform models, where the GAN was trained on models of similar form to that in (**a**). Note that the horizontal axis represents horizontal distance between the transects.

Training a GAN to produce images of space-time transforms such as that in Fig. 1b is impractical because the important (coloured) parts of the image change location within the panel for each GPM run. Therefore, transforms are first converted to a form where the vertical axis represents time of deposition and the colourmap represents the corresponding height on the vertical transect through the geological model at horizontal offset *x*. All of our GPM simulations have a time span of 5 Ma so all features of interest then span the entire vertical axis. Figure 3a shows an example transform which corresponds to that in Fig. 1b.

### Generative adversarial network

A Generative Adversarial Network (GAN) is a mathematical construct from the machine learning community which uses a neural network called a Generator to create samples of a probability distribution that is trained to emulate some target distribution. In our case the target distribution represents the set of geological transforms produced by GPM given prior information about active geological processes. Traditional machine learning techniques would use a mathematical formula to evaluate whether generated samples resemble samples of the target distribution. A GAN however uses a separate neural network for this evaluation—the so-called discriminator *D* which is trained simultaneously with the Generator *G*.

During the training phase of the GAN, *D* is fed samples from both the target distribution and from *G*. *D* is trained to discriminate from which distribution each sample originates. *G* is trained to generate samples that are indistinguishable from target distribution samples—in effect it is trained to ‘fool’ the discriminator into predicting that its samples are directly from the target distribution. Thus, *D* and *G* have adversarial objectives and training can be difficult as both networks must be effective for either to be so. In this work we use the WGAN-GP network^37^ that improves upon the original GAN by introducing a more effective loss fuction. The loss functions for *D* and *G* respectively are:

1 $$\begin{aligned} L_D = \mathbb {E}_{\tilde{m}\sim \mathbb {P}_G} [D(\tilde{m})] - \mathbb {E}_{m\sim \mathbb {P}_{GPM}} [D(m)] + \lambda \mathbb {E}_{\hat{m}\sim \mathbb {P}_{\hat{m}}} [(||\nabla _{\hat{x}}D(\hat{m})||_2-1)^2] \end{aligned}$$

and

2 $$\begin{aligned} L_G = - \mathbb {E}_{\tilde{m}\sim \mathbb {P}_G} [D(\tilde{m})] \end{aligned}$$

where *m*, $\tilde{m}$, and $\hat{m}$ are respectively models from the GPM, models generated by *G*, and model samples taken randomly from either distribution, and where $\lambda$ is a gradient penalty weighting parameter which is generally set to 10.

Our implementation of the WGAN-GP network is based on^40^ and on numerous tests to find a structure that performs effectively. Specifically, the generator consists of five blocks each with three convolutional layers, each preceded by batch normalisation layers. The discriminator consists of 6 blocks each with three convolutional layers followed by a pooling layer which reduces the dimensionality. The blocks gradually reduce the number of features upon which they act such that the first block influences large areas and the last block controls the finer details. The latent space consists of 30 independent Gaussian distributed parameters which map to generated height-to-depth conversion models of 128-by-128 parameters.

### Estimating correlations and tomographic images

Consider a scenario where multiple geochemical logs are recorded along vertical transects through the same sedimentary basin, and assume that the geochemical signatures observed in logs originate from secular variations in seawater chemistry. If the correct height-to-time transform is applied to any log, each true $\delta ^{13}$C variation with respect to time should be revealed, other than during periods of hiatus; conversely, the same log will usually exhibit an erroneous secular signal if an incorrect transform is applied. As a result, only the correct transform will map all logs onto an identical secular signal within overlapping time periods. The match between transformed data on each log can therefore be used as a diagnostic of the quality of any space-time transform.

The misalignment or misfit between any pair of transformed logs can be quantified by an L_2_-norm misfit measure:

3 $$\begin{aligned} S := \sqrt{\frac{1}{N} \sum _{i=1}^{N} (x_i - y_i)^2} \end{aligned}$$

where $x_i$ and $y_i$ are the *i*-th time samples predicted from the two logs, and *N* is the number of sample pairs. In order to evaluate Eq. (3) both logs must have the same sampling in time, so each log is first interpolated to match the time sampling of the other resulting in two comparable pairs of logs. Squared misfits are calculated for both interpolations, and all results are summed in Eq. (3) before the square root is taken. When multiple pairs of logs exist, each log should first be interpolated onto the time sampling of each of the others, and the misfits between all pairs are summed; the result is then divided by the total number *N* of sample pairs, after which the square root is taken.

While the relative location of the transects to each other is known, the absolute lateral location relative to any geological model is not. We therefore first shift the set of transects laterally across each model sample to find the lowest misfit value according to Eq. (3) for that model, and fix the horizontal location of the transects relative to the stratigraphy at the location of that minimum.

Given the nonlinear nature of space-time relationships and the fact that measured data contain errors, different transforms may yield potentially satisfactory correlations. Bayesian inversion addresses this possibility by characterising the distribution of all possible transform models given the log data. This distribution is known as the posterior probability distribution function (pdf), which we refer to herein simply as the posterior. This can be calculated by evaluating Bayes rule

4 $$\begin{aligned} \rho (\varvec{m}|\varvec{d}) = \frac{ \rho (\varvec{d}|\varvec{m}) \rho (\varvec{m}) }{ \rho (\varvec{d}) } \end{aligned}$$

where $\varvec{m}$ defines a space-time transform such as that in Fig. 3a, $\varvec{d}$ is a vector of observed log data, $\rho (\varvec{d}|\varvec{m})$ is called the data likelihood (a non-normalised pdf that describes the data fit provided by transform $\varvec{m}$), $\rho (\varvec{d})$ is the evidence which is constant for fixed data observations $\varvec{d}$, and $\rho (\varvec{m})$ is the prior distribution which contains information known about the transform independently of the current dataset. In this study, geological information within the GAN is used as the prior pdf and represents information within the set of models used to train the GAN. This constrains the posterior distribution to models that resemble potential results from GPM simulations.

We simulate transform models $\varvec{m}$ from the posterior distributions using a Metropolis-Hastings Markov-Chain Monte Carlo (McMC) method. McMC samples the posterior distribution by creating chains of samples, where a new sample $\varvec{m}'$ in the chain is related to the preceding sample $\varvec{m}$ by a proposal distribution $q(\varvec{m}'|\varvec{m})$ and is accepted with probability:

5 $$\begin{aligned} P_{accept} = \left\{ 1, \frac{\rho (\varvec{m}')\rho (\varvec{d}|\varvec{m}')q(\varvec{m}|\varvec{m}')}{\rho (\varvec{m})\rho (\varvec{d}|\varvec{m})q(\varvec{m}'|\varvec{m})} \right\} \end{aligned}$$

If sample $\varvec{m}'$ is not accepted then $\varvec{m}$ is repeated as the current sample of the chain. The density of model samples in the chain is proven to converge towards the true posterior distribution when the number of samples tends to infinity^41,42^. In our case the proposal distribution is defined to be Gaussian, and by creating multiple chains with different random initial models drawn from the prior distribution we create an ensemble of chains whose samples together converge towards the posterior distribution more rapidly.

The McMC algorithm does not sample the space-time transforms similar to Fig. 3a directly, but rather the low-dimensional latent space of the GAN. Thus, each sample proposed by the McMC algorithm is mapped to a space-time transform model by the GAN, which in turn is used to convert logs to time in order to calculate misfit *S* according to Eq. (3). Finally, Eq. (5) can be evaluated by assuming Gaussian uncertainties with unit variance on the log data, resulting in a likelihood proportional to $\exp (-S^2)$. In real-data examples the variance would be determined by expected geochemical laboratory measurement errors. The sample is accepted or rejected according to Eq. (5), allowing the chain to progress.

## Results

**Figure 4:**
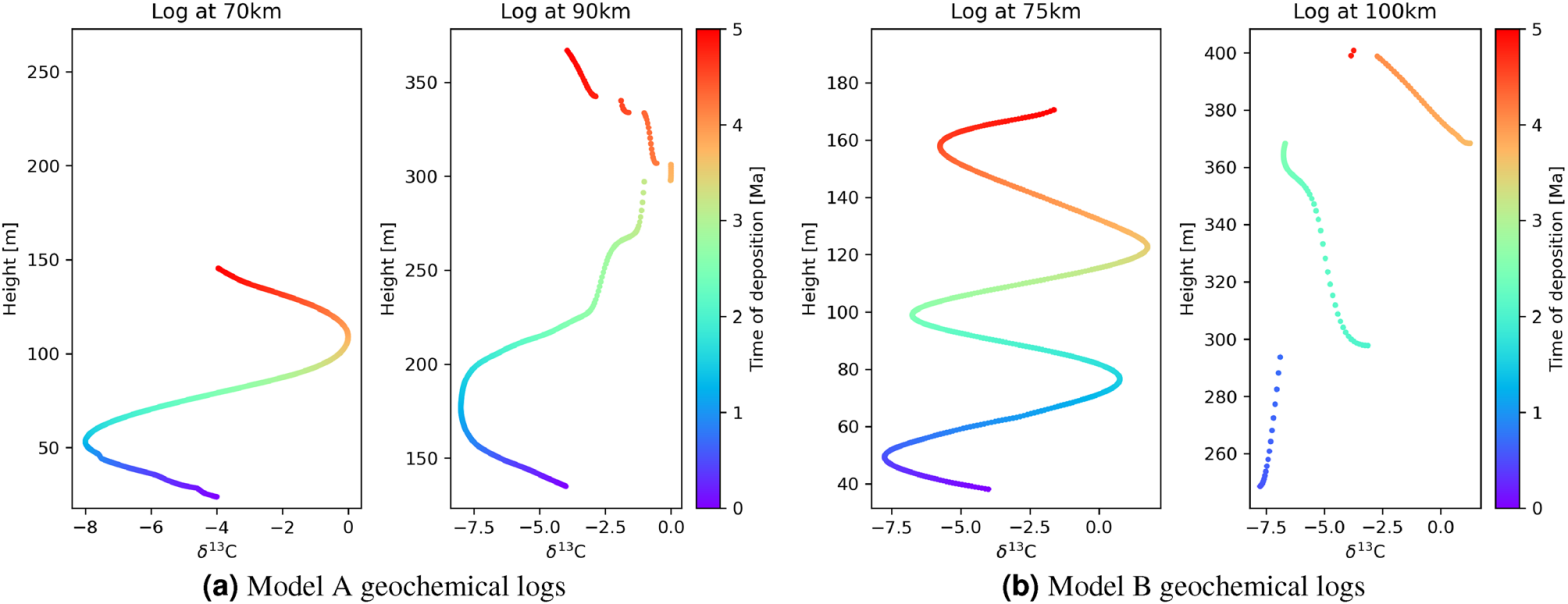
Simulated logs on vertical transects for: (**a**) geological model A with logs at lateral locations 70 km and 90 km, and (**b**) geological model B with logs at lateral locations 75 km and 100 km, as shown in Figs. 1 and 2, respectively. Colours correspond to the age of deposition of preserved sediment and adhere to the colourmap of Figs. 1b and 2b.

Assuming the purely secular geochemical signature shown in Fig. 1b, geochemical sampling along the two transects in Fig. 1 produces the synthetic logs in Fig. 4a. Each log consists of $\delta ^{13}$C ratios in a preserved sediment at each time step of 10k years, providing a median spatial sampling interval of 0.26 m. This sampling density accounts for the apparent smoothness of the logs, and also for the individual points visible around 300 m height in the right hand log. While this sampling is relatively dense compared to many field campaigns^43,44^, some field^45,46^ and core^47^ related sampling schemes are similarly dense. Due to differences in sedimentation rate between the two locations, the log at 70 km is spatially compressed compared to that at 90 km. Furthermore, while the deeper water (left-hand) logs show an almost complete geochemical signature in the sense that they reflect most of the secular variation, the shallower logs exhibit hiatuses (reflected in apparent discontinuities) down to about 300 m in height, caused by erosion of deposited material.

For data collected in the field the age of deposition of each sample is rarely known. An approximate age may be estimated for samples which lie within a dateable facies types^48^, and geochemists typically interpolate the ages of other samples between these facies. Similar temporal patterns are sought within different logs in order to correlate between transects, despite errors in interpolated ages. In the case of shallow marine sediments, these errors can be expected to be large, and may distort geochemical signatures unrecognisably^6^.

The true time of deposition is known for the modeled logs in Fig. 4a and is illustrated by the colour scale; correlation between the transects consists of matching colours between the logs. Due to the simplicity of the sinusoidal geochemical signature which is clearly visible on both transects, these data would be relatively straightforward to correlate even if these were field data without known times of deposition. However, this is not generally the case for more complex secular geochemical variations or different geological models, for example as shown in Fig. 4b. In that case, if the time of deposition was unknown it would be difficult to make an accurate unique estimate of the true correlation. Instead, the family of all possible correlations should be interpreted if scientific inferences are to be robust.

Representative examples of space-time transform models produced by the GAN are shown in Fig. 3b. Models in the prior distribution are oriented both with the shallow region on the left and the right, assuming that *a priori* we do not know the orientation of the geological structure relative to the transects. Other than this left/right ambiguity, model samples show similar features to the example in Fig. 3a albeit at slightly reduced definition, indicating that the GAN represents space-time transforms to a reasonable level of detail.

**Figure 5:**
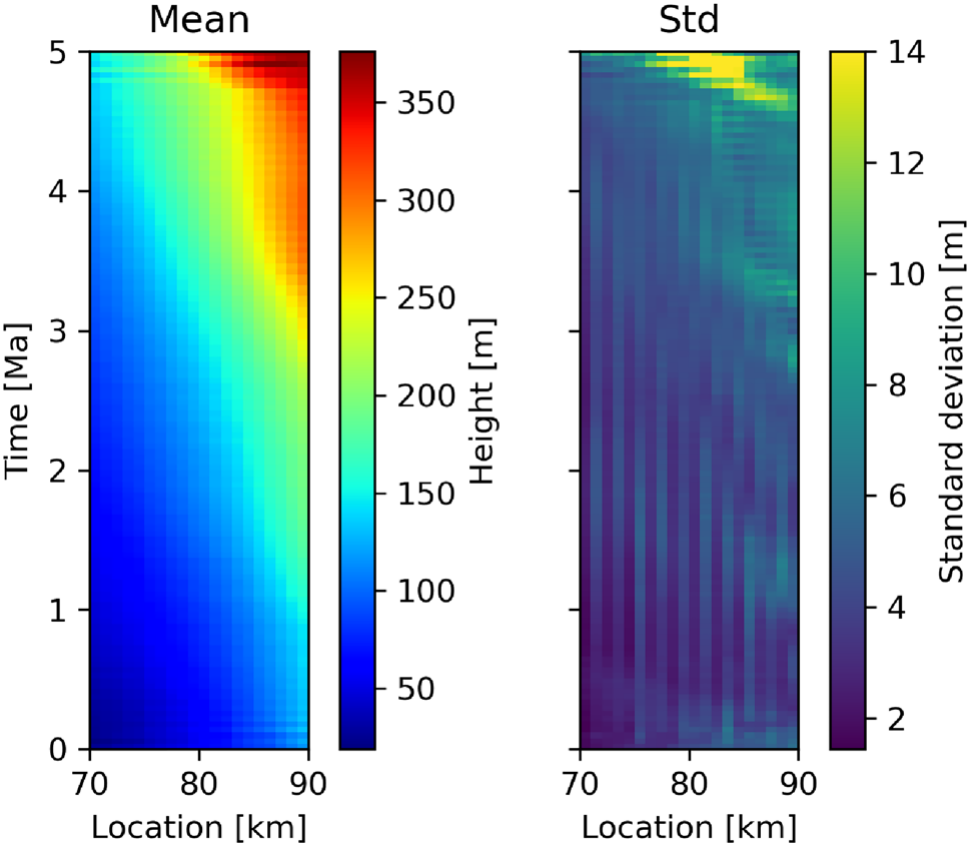
Mean and standard deviation of the posterior space-time transforms for geological model A calculated over all posterior samples.

The horizontal axis of GAN generated transform models represents distance between the transects, whereas the geochemical logs are measured at absolute locations. GAN generated transform models are translated to the absolute location-axis by finding the best-fit horizontal locations of the logs within each GAN model by minimising *S* in Eq. (3). The posterior distribution is then generated according to Eqs. (4) and (5), and the family of posterior transforms can be characterised using various statistics such as the posterior mean and standard deviation at each point in the space-time transform (Fig. 5). The left and right logs are shown to have mean height ranges of 25–150 m and 140–360 m respectively (colours, left panel of Fig. 5) and span the full 5 Ma period, which is consistent with the true deposition shown in Fig. 1b. Standard deviations (right panel) are small throughout the model with a higher uncertainty between 3 Ma and 5 Ma at the right edge which corresponds to the locations of hiatuses in the right-hand log observed in Fig. 4a, and that ambiguity is shown to create a specific locus of intense uncertainty in the space-time transform around 80 km at 4.9 Ma.

**Figure 6:**
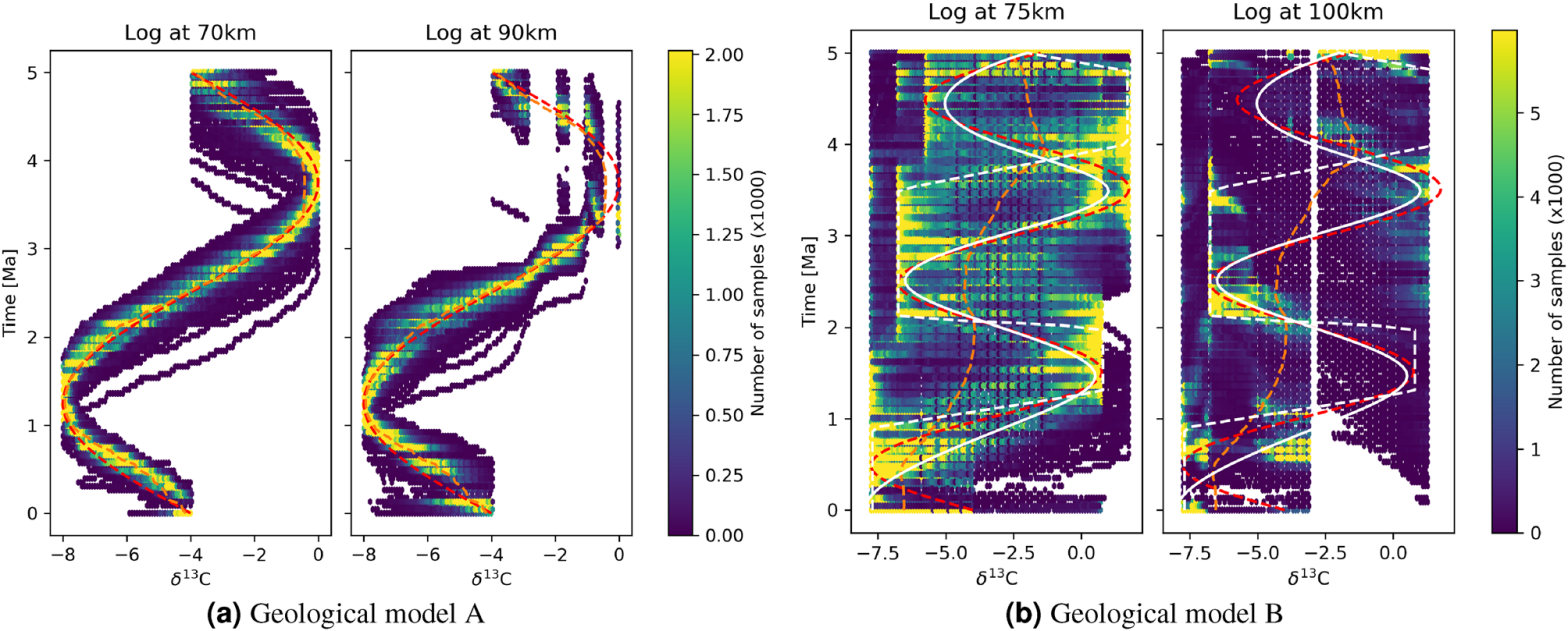
Logs through (**a**) geological model A logs (Fig. 4a) and (**b**) geological model B logs (Fig. 4b) converted from height to time using 10,000 posterior transform samples, represented as a histogram of number of samples predicting each $\delta ^{13}$C value at each time. Lighter colours indicate a greater number of posterior samples are mapped to the same location in the panel. Red dashed line is the true geochemical signature; orange dashed line indicates the posterior mean geochemical signature. Solid and dashed white lines in (**b**) represent two high-probability posterior geochemical trends analysed further in the main text.

If the correct space-time transform is chosen then the two logs should map to the same geochemical signature in time. Figure 6a shows the histogram of logs through geological model A in Fig. 4a converted to time using 10,000 posterior samples of space-time transforms, while the red line indicates the true geochemical signature. Lighter colours indicate that more samples mapped logs to those locations of the plot, which indicates higher posterior probabilities that geochemical samples were deposited at those times. There is a close resemblance between high probability regions of both logs and the true geochemical signature, indicating that we find approximately correct models for these logs. We also note for later that the uncertainty around high probability areas is approximately symmetrical in this case. The vertical lines around 4 Ma for the right log indicate missing data during hiatuses, as seen in Fig. 4a.

**Figure 7:**
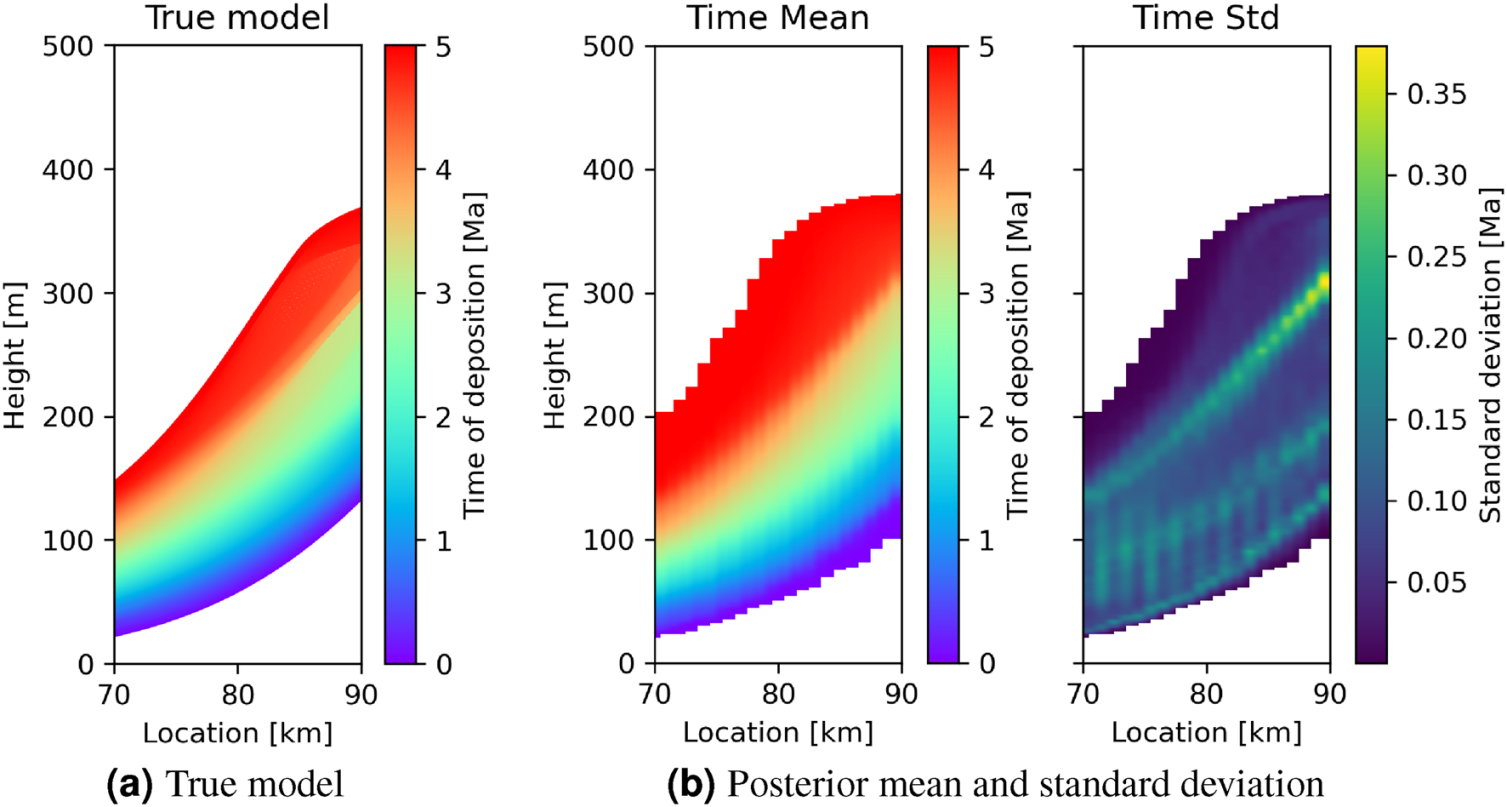
(**a**) True time of deposition in geological model A, and (**b**) posterior mean and standard deviation of the time of deposition for locations between the transects in Fig. 1.

The analyses above only considers height-time transforms at the location of the logs, yet these transforms are also defined between the log locations as seen in Fig. 5. This allows us to perform inter-transect geochemical tomography: by switching the time of deposition and height axes in each model sample, then recalculating the mean and standard deviation, in Fig. 7b we show a cross-section that corresponds to that through the true model in Fig. 1b and repeated in Fig. 7a. The mean model includes additional deposition close to 0 Ma and 5 Ma indicated by the slightly thicker red and dark blue areas at the top and bottom, presumably because the data do not adequately constrain rates or durations of deposition close to the temporal boundaries of the data. These regions show low uncertainties due to the fact that the height-to-time conversion models were all drawn from a prior distribution that fixed the geological simulations to be between 0 and 5 Ma, which is appropriate if, for example, dateable facies occur at top and bottom of the succession. Intuitively, if any sediment exists in the uppermost parts of the model then it must have been deposited close to 0 Ma, with low uncertainty on that interpretation, and similarly for lowermost areas. The step changes in true age caused by erosion in the true model around 300 m height are less pronounced in the mean model, but in the map of standard deviation these boundaries cause the appearance of so-called uncertainty loops (regions of high uncertainties spanning portions of the model that exhibit rapid lateral changes, surrounding regions which are relatively well constrained^49^). These loops appear because perturbations in the location of a discontinuity in model parameter values have little effect on the data, and indicate that the exact location of such discontinuities remains uncertain. The geometrical configuration of uncertainty loops is itself an interpretable indicator of a potential discontinuity, as has been observed in other types of studies (seismic tomography^49^, electrical resistance tomography^50^, ambient-noise tomography^51^, and grain orientations in anisotropic media^52^). This is the first time that this phenomenon has been observable using geochemical data.

Compared to model A, correlation between the logs through geological model B in Fig. 4b is significantly more difficult because hiatuses in the shallow log at location 100 km cause large temporal discontinuities. We apply the Bayesian correlation and tomography scheme to convert the logs to the time domain, and the histogram of results from 10,000 posterior transform samples is illustrated in Fig. 6b. Brighter areas represent places to which log samples are more often mapped in time, and the red dashed line is the true geochemical signature. While there is a greater spread in the deeper (left-hand) log compared to that in the simpler case in Fig. 6a, we see that higher probability areas in both logs tend to coincide with the true geochemical signature. Due to the large data loss to hiatuses, the shallow log exhibits fewer high probability areas, but most of them span the true geochemical signature. However, while in Fig. 6a the probability of $\delta ^{13}$C was centrally peaked around the mean which also coincided approximately with the true secular variation, in Fig. 6b there appear to be at least two high probability secular change curves shown in white (solid and dashed). This indicates multimodality in the posterior solution (that is, there are separate regions of the space of possible transforms which have a high posterior probability), and as expected the mean (orange curve) then lies between the high probability solutions and has a *low* probability of being true.

**Figure 8:**
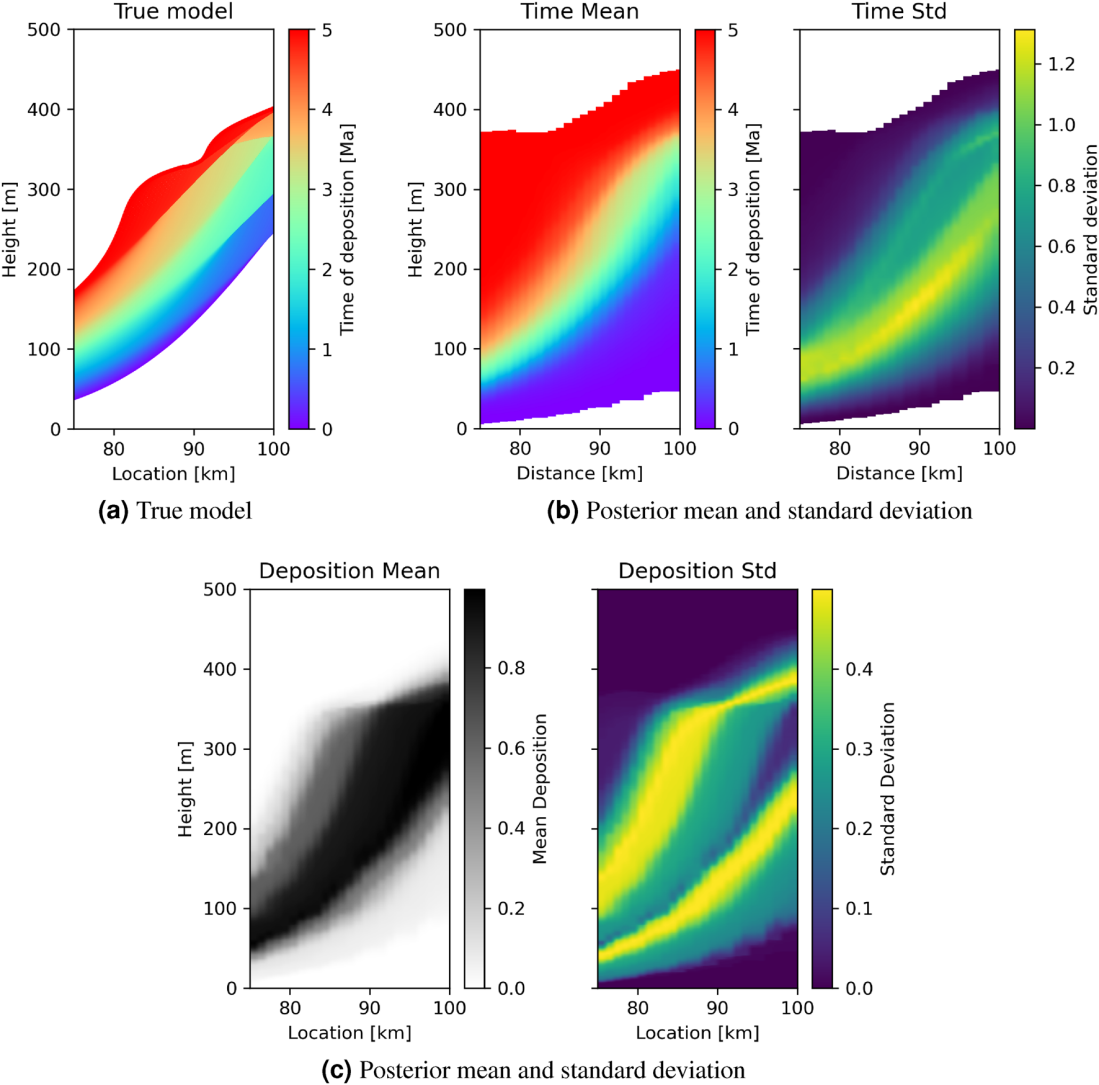
(**a**) True time of deposition in geological model B, and (**b**) posterior mean and standard deviation of the time of deposition for locations between the transects in Fig. 2. (**c**) Posterior mean and standard deviation of whether sediment is present at that location (value 1 indicates presend, 0 indicates absence).

Figure 8 shows the true time of deposition (panel (a)) and the posterior mean and standard deviation of the tomographic results (panel (b)). Similarly to the less complicated scenario, the mean model resembles the true model but in this case discontinuities in time are not as well defined. We also observe that the light blue time period is displaced from its true location, but note that the results alert us to this possibility through high uncertainties for that space-time interval. The standard deviation map shows uncertainty loops around the locations of hiatuses; this shows that the posterior distribution contains information about hiatuses and provides explicitly quantified uncertainty about their exact spatial locations and durations. And similarly to the results for Model A, the posterior mean extends the depositional areas around 0 Ma and 5 Ma (bottom right and top left, respectively), again because the data only poorly constrain deposition duration and rates close to the start and end of the time period considered. Since the mean and standard deviation maps can only be calculated across models in which sediment was deposited at each location, panel (c) shows additionally the posterior mean and standard deviation of whether sediment is present at each location (value 1 indicates presence, 0 indicates absence). This data shows the constraints that the transect data place on exactly where sediment was deposited within the 5 Ma period, and exhibits uncertainty loops around the edges as expected. The combined posterior results in panels (a) to (c) thus provide quantitative constraints on both time of deposition and delineation of the sedimentary domain corresponding to the age range of interest.

**Figure 9:**
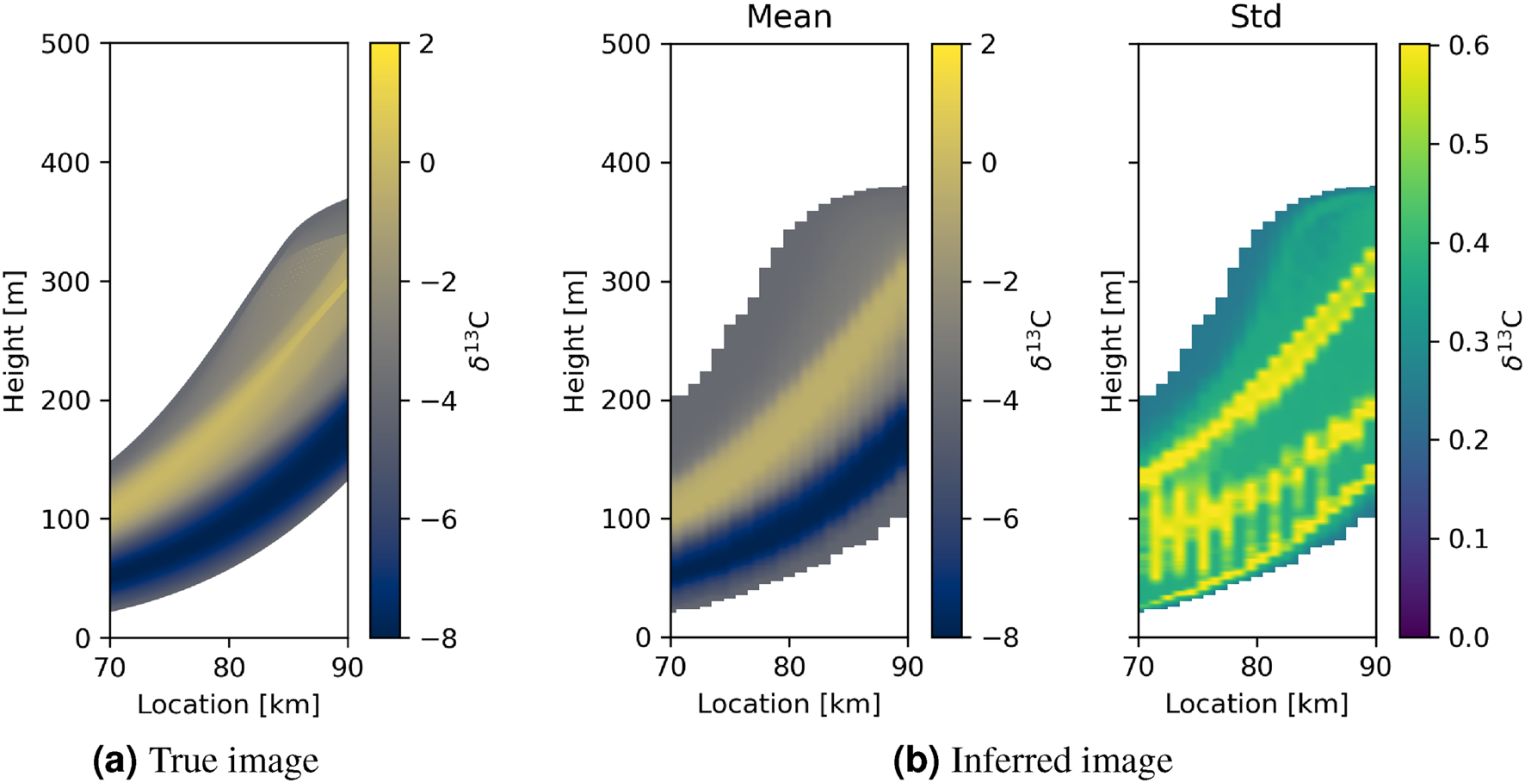
Tomographic images of geochemical signatures of sediments between the transects through geological model A. (**a**) shows true $\delta ^{13}$C values, and (**b**) the inferred mean image and standard deviation. Image (**b**) is constructed from the mean and standard deviation time of deposition models in Fig. 7b and an estimated mean and standard deviation geochemical signature derived from Fig. 6a.

One intriguing aspect of the tomographic models is that they can be combined with the inferred secular change curves to predict geochemical signatures of sediments across the tomographic image. Figure 9 shows the resulting true and inferred inter-transect geochemical images corresponding to geological model A. The inferred geochemical values are estimated using the mean inter-transect time of deposition in Fig. 7b, by estimating the mean geochemical variation from Fig. 6a. Overall, there is good resemblance between the true and inferred geochemical values, but also less pronounced discontinuities, and extensions outside of the true region of deposition due to the poor constraint on depositional durations at the top and bottom of the formation. These features are inherited because this result is a combination of the results in Figs. 7b and 6a which show similar features.

**Figure 10:**
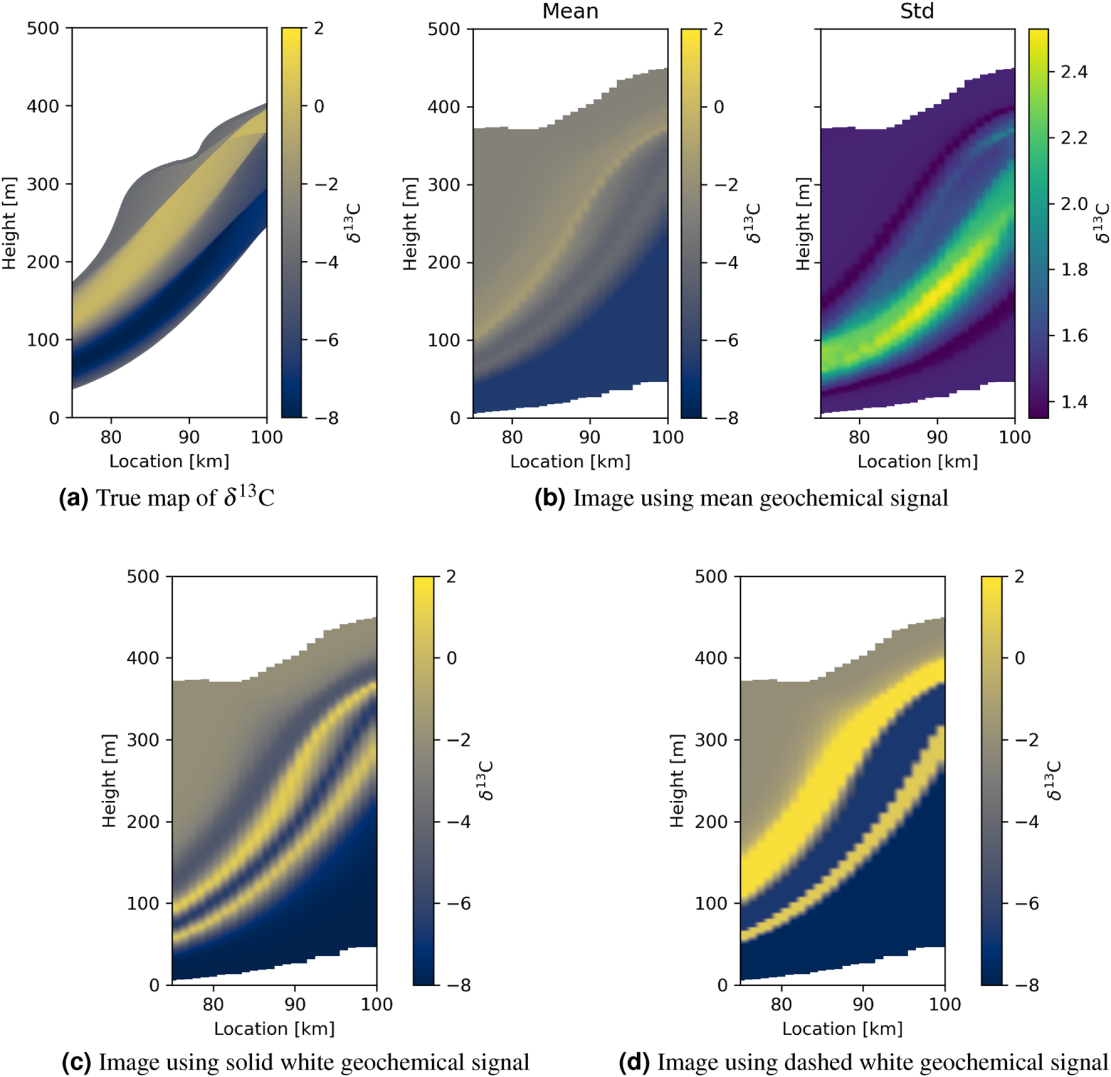
Tomographic images of geochemical signatures of sediments between the transects through geological model B. (**a**) shows the true distribution of $\delta ^{13}$C values, and (**b**–**d**) the inferred images. The inferred images are constructed from the mean and standard deviation time of deposition model in Fig. 8b and estimated secular variations derived from the mean, the solid white and the dashed white geochemical signatures in Fig. 6b for (**b**–**d**), respectively.

For geological model B, the true and inferred inter-transect geochemical images constructed from the mean model of Fig. 8 and the mean of Fig. 6a are shown in Fig. 10b, and exhibit far less resemblance than for case A. The geochemical signature in Fig. 6b is not recovered well, reflecting the fact that the mean of Fig. 6b is in fact a poor indicator of the true change in $\delta ^{13}$C . The lower panels in Fig. 10 show images constructed using each of the two interpreted high probability $\delta ^{13}$C signals (white lines) in Fig. 6b. In panels (c) and (d) the band of high $\delta ^{13}$C is better resolved compared to when using the mean geochemical signal in panel (b). However, both (c) and (d) show a second high $\delta ^{13}$C area near the bottom of the image that is not present in the true image. This is explained by Fig. 6b in which neither of the interpreted curves fit the true secular variation close to 0 Ma - and indeed that the true variation is impossible to constrain accurately around this time interval because information is missing around the red curve due to depositional hiatuses. This proves first, that accounting for uncertainty in the correlation of the logs is critical to represent the final state of knowledge about the geochemistry of the formation. Second, that individual modes of the posterior probability distribution function describing uncertainty in the correlation must be accounted for separately, rather than using statistics such as the mean and standard deviation which combine the information from multiple modes under the assumption that the underlying posterior probability distribution is centrally focused—which is incorrect in this case.

## Discussion

This work advances geochemical correlation methods by deploying Bayesian methods to evaluate uncertainties in the results. It also introduces constraints from geological prior information, and estimates a family of possible secular $\delta ^{13}$C variations, producing tomographic images of both the time of deposition and the geochemical signatures of sediments in the space between geochemical sampling transects.

There are distinct complications and implicit ambiguities in the interpretation of geochemical records from shallow marine environments^6^. Figures 6b and 10 illustrate clearly that irreducible and complex uncertainty remains in the correlation between logs, even with the addition of geological prior information. The importance of analysing the full Bayesian uncertainty in a sensible way, rather than simply using mean models has been recognised in studies that use manual qualitative correlation methods (e.g.,^11^ suggested four possible correlations of Ediacaran to Cambrian global geochemical records). Our quantitative method exhibits these uncertainties explicitly, mitigating against the effects of scientific overconfidence and other interpretative human biases^53–56^ that often lead to herding behaviour^57^. This is critical for subsequent research that relies on the results of correlation studies: for example, high-precision U-Pb zircon age constraints on the end Permian of West Texas, have required dramatic modifications in the interpreted durations of discernible sedimentary packages, and therefore in the inferred rates of sea-level change and biological events such as rates of mass extinction, as can be seen by comparing^58^ with^59^.

Whether manual or automated, a weakness of previous correlation methods is that provided the sampling on each transect is already sufficient, correlation results cannot be formally tested against further independent data sets of similar type. If a new transect is sampled then its log merely needs to be correlated with the existing, already correlated logs. While in some cases this might lead to alternative hypotheses for the original correlations (e.g.,^60^), the original correlations can rarely be refuted, and their relative likelihood of being true cannot be evaluated. This is because uncertainties in the new correlation are of similar type and magnitude to those already incurred. By contrast, our method is quantitatively testable: each posterior distribution of correlations implies an inferred distribution of tomographic age models for the inter-transect space. These can in turn be converted to a posterior distribution of maps of inter-transect geochemical signatures: the mean inter-transect geochemical signatures are shown in Fig. 9, and both the mean and modal solutions are shown in Fig. 10, for geological models A and B, respectively, all of which form testable hypotheses of the corresponding space-time correlations. A further transect could subsequently be sampled in the inter-transect space, the data from which would allow a quantitative significance test of the robustness of each hypothesis, directly corresponding to a test of the original inferred distribution of inter-transect correlations.

Current methods use pattern matching to correlate geochemical logs, and so can be foiled due to hiatuses caused by erosion and by changes in sedimentation rates which distort observed geochemical signatures^8^. Provided that appropriate geological process models are used for the geology being sampled, our method should be more robust in such situations because prior information about the dynamic geological processes is used to constrain the family of possible nonlinear distortions. Correlation is performed in the time domain using the corresponding family of possible space-time transforms to undo these distortions, and the correct transform projects all observed logs to the same time-axis. In cases where geochemical data are mainly controlled by secular change, the similarity of disparate logs in that domain is a measure of the quality of any particular space-time transform and its implied inter-transect correlation.

Our tests of this method involve geochemical sampling densities far higher than are often performed in the field. Typical sampling densities are approximately one sample per meter, whereas in comparison the samples used herein are effectively continuous over much of the time period. These tests therefore illustrate results for an effectively optimal sampling scenario, yet they demonstrate that significant uncertainties remain in inter-transect correlations. This corroborates the findings of^6^ who explained why even infinitely dense data from shallow marine environments cause humans to correlate logs erroneously due to hiatuses and other effects. We show that Bayesian inversion is nevertheless able to quantify the resulting uncertainty in correlations, and to provide tomographic estimates of the region between transects.

A limitation of our method is that the GAN distribution is limited to samples that resemble the GPM models. In our tests the GPM models represent a widely varying but nevertheless geologically limited set of models, and therefore the prior distribution only represents this limited variation in geological models. The GAN distribution is a manifold (hyper-surface) within a higher dimensional space, thus any true model outwith this manifold may not be represented precisely by the GAN, and in turn may only be approached by the inversion but never found exactly. This problem occurs for any method of parametrization and in any inversion scheme, and therefore equal care has to be taken to train a GAN that represents a wide variety of geological models.

Different generative networks exist that can achieve similar or perhaps even improved generational quality such as diffusive models^61^, or which provide uncertainties on their generated samples such as Bayesian Flow Networks^62^. However, with increased complexity and diversity within the training set, more training data is required to ensure that the prior model space is accurately sampled. Thus, there is a trade-off between the complexity and diversity of the generated samples, and the amount of training data and cost of training. We opted for a more established GAN where training is well understood at the expense of potentially reduced variability in the generated samples. Future work may explore different network types and increased generational flexibility.

The posterior distributions characterised herein are all statistical inferences, except for the lateral locations of logs relative to the model samples which is approached as an optimisation problem. This is not obligatory and could itself be implemented as a sampling process. Optimisation was chosen purely for computational efficiency. While GANs are faster than running the GPM, their computational speed depends on the hardware on which they are running. Graphics Processing Units (GPU) run convolutional Neural Networks, like the GAN, an order of magnitude faster than Central Processing Units (CPU)^63^. McMC inference results herein use CPUs, so we invoked this optimisation method to improve performance.

Various extensions of this work are possible. Geochemical proxy values are also influenced by factors such as the signal preservation properties of different facies^1^, the seawater depth at time of deposition^64^ and post-depositional diagenetic effects^65^. These factors have not been included explicitly in this work, but can be introduced similarly, simply by including them in the GPM. For example, by recording water depth at time of deposition from the GPM results, geochemical water depth dependencies could be modelled and accounted for in the transforms used to project data to the common time domain. The consistency of projected data could then be used to discriminate between different models of depth control, using panels analogous to those in Fig. 6. Further emphasis could then be placed on testing this method in conjunction with true models that are inconsistent with the geological prior information introduced. Theoretically, provided the prior information does not categorically exclude the true structure, Bayesian methods should be able to convert the prior distribution into a posterior distribution that includes that true result. In practice however, Eq. (4) shows that the choice of prior distribution has significant influence on the resulting posterior distribution, and in turn impacts the ability of algorithms such as Monte Carlo methods to find models that lie close to the truth. In the extreme case where the true structure has zero prior probability the inversion can only approach the true model but never find it. To mitigate against this, the prior distribution should span a broadly conceived range of scenarios consistent with a variety of geological concepts, such that the zero probability region will reduce to non-geological models only. As shown by^30^ using seismological rather than geochemical data, it may then even be possible to use geochemical data to discriminate between conceptual geological models, excluding those that are inconsistent with the true structure.

Lastly, since the GPM models in this study were all 3-dimensional from which we selected 2-dimensional cross-sections for illustration, it is straightforward to apply the method to find 3-dimensional inter-transect images. Indeed, while we have only illustrated tomographic models between the transect pairs, the images can be extended outside of this volume, with lower accuracy. It is even possible to image the volume (without performing correlation) using data from a single transect, in effect implementing the example in^66^ tomographically.

## Conclusion

Current geochemical correlation methods are designed to match patterns in data observed on different transects to find samples that appear to correspond to the same geological time of deposition. However, the relationship between the log height and time of deposition is highly nonlinear due to differences in local sedimentation rates and hiatuses in the data due to erosion or non-deposition of sediments. A novel, semi-automated method to correlate geochemical logs using Bayesian inference with geological prior information yields correlations with statistical uncertainties, and constructs tomographic images of the time of deposition or geochemical signatures in the space between the transects. The method finds correlations and tomographic images even in complex scenarios where pattern matching methods break down, and allows correlation and secular change hypotheses to be tested against subsequent independent data sets of the same type, promising significant advances in quantitative statistical inference from geochemical logs.

## Data Availability

Data for this work has been generated using SedSimple which is available for free from Westchase Software Corporation. Configuration files for the SedSimple runs are available upon request.
